# Supplementary material for: 4.2 K sensitivity-tunable radio frequency reflectometry of a physically defined p-channel silicon quantum dot
Source: Sci Rep. 2021 Oct 8;11:20039. doi: 10.1038/s41598-021-99560-x (PMC8501031; doi:10.1038/s41598-021-99560-x)
Supplement: Supplementary file 1 — Supplementary Information. [file 41598_2021_99560_MOESM1_ESM.pdf]

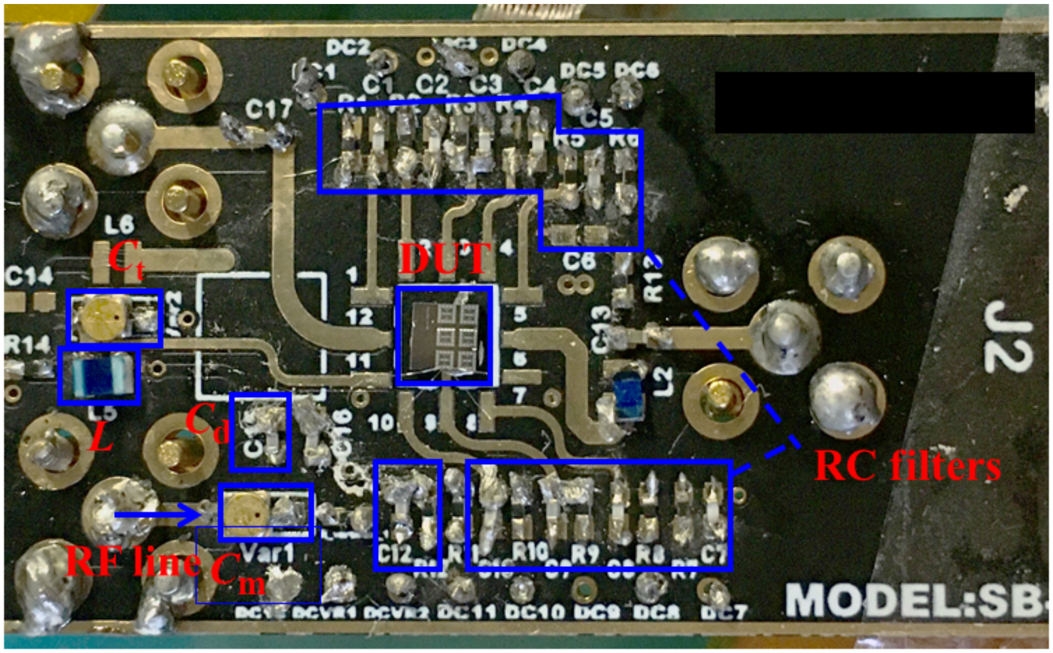

Figure 1. FR4-type PCB used in the measurement with the device under test (DUT) at the central part.

SUPPLEMENTARY INFORMATION

S1. Printed Circuit Board (PCB)

In this study, we used a FR4 type PCB (Fig. A1) in which two voltage-tunable capacitors (a Macom MA46H202-1056 for C_m and a Macom MA46H204-1056 for C_t) are connected in parallel to a series RLC impedance transformer circuit having a non-magnetic inductor of 277 nH, and a mounted chip comprising six pMOS devices (DUT). Only two devices (bottom and middle left) are used in these experiments. Connections between the devices and the readout circuit are made by bond wires. Both varactors voltages, respectively V_m and V_t, were filtered by single stage RC filters (R =10 kΩ, C =270 pF) with a cut-off frequency of 60 kHz. The same filters were also applied to the device biases.

S2. Impedance matching

For impedance matching purposes, we first simulated our circuit using National Instrument AWR software and determined the values for C_m and C_t that gave a maximum amplitude for ΔS_{11}. These values were then checked against experimental ones by using the converting tables between the varactor voltages and the capacitance values that are available from Macom. In Fig. A2, perfect impedance matching was achieved for $C_\mathrm{t} \sim 6$ pF (V_t = 5 V) and $C_\mathrm{m} \sim 5.5$ pF (V_m = 1.5 V) from data sheet of varactors. The reflection coefficient $\Gamma = (Z_\mathrm{L} - Z_0)/(Z_\mathrm{L} + Z_0)$ where Z_L is the circuit load impedance and Z_0 = 50 Ω allows checking the optimum values obtained above and leading to a perfect impedance matching.

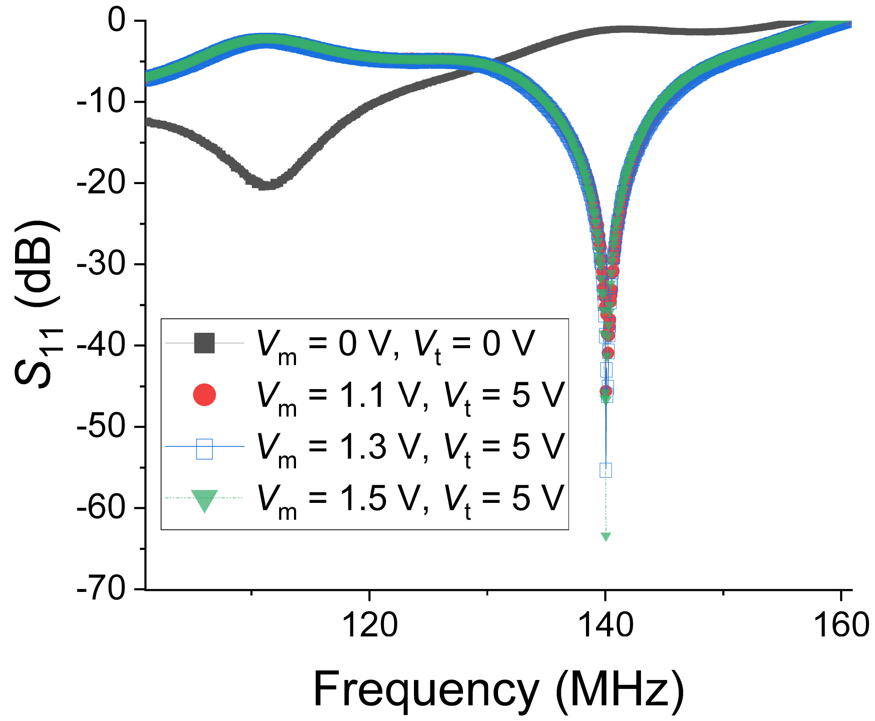

Figure 2. Reflected signal S_{11} measured at different values of the varactor voltage V_m with device 1. The first curve at $V_t = 0$ is shown as a reference. Perfect matching is obtained when $\Gamma = 0$. From measurement data, we found $|\Gamma| \sim 0.005$ meaning near perfect matching was achieved experimentally.
